# Meetings of American Dental Convention, Southern Dental Association, Etc.

**Published:** 1877-08

**Authors:** 


### ARTICLE IV.

### *Meetings of the American Dental Convention and other Dental Associations.*

The Annual Meeting of the American Dental Convention, and the Dental Association of Maryland and District of Columbia, and a Special Meeting of the Southern Dental

Association, will be held at Deer Park and Oakland, Garret County, Maryland, commencing Tuesday, August 14th, 1877, at 11 A. M., and continuing through the week.

This is to be a united, but not a consolidated meeting of the three Associations, for while it will consist of a series of joint sessions, each body will control its own internal affairs, and each individual will preserve his distinctive membership in his own body, and at his option acquire membership in the others.

The sessions will be held in the Memorial Church in Oakland, and members will have conveyance at suitable hours between the two places.

American Dental Convention.

*President.*—W. H. Atkinson, New York.
*Vice-President.*—J. R. Walker, New Orleans.
*Cor. Secretary.*—G. A. Mills, Baltimore.
*Rec. Secretary.*—Ambler Tees, Philadelphia.
*Treasurer.*—J. G. Ambler, New York.

*Executive Committee.*—R. B. Winder, Baltimore; H. Townsend, Philadelphia; W. M. Reynolds, New York; G. A. Mills, Baltimore; L. H. Delange, Camden, N. J.

Southern Dental Association.

*President.*—S. J. Cobb, Nashville, Tenn.
1*st Vice-President.*—A. C. Ford, Atlanta, Ga.
2*nd Vice-President.*—A. F. McLain, New Orleans, La.
3*rd Vice-President.*—J. B. Patrick, Charleston, S. C.
*Cor. Secretary.*—W. L. Dismuke, Nashville, Tenn.
*Rec. Secretary.*—E. S. Chisolm, Tuscaloosa, Ala.
*Treasurer.*—H. A. Lowrance, Athens, Ga.

*Executive Committee.*—L. D. Carpenter, Atlanta, Ga.; J. G. McAuley, Selma, Ala.; J. G. Angell, New Orleans, La.

Dental Association of Maryland and District of Columbia.

*President.*—R. Finley Hunt, Washington.
*Vice-President.*—B. F. Coy, Baltimore.

*Cor. Secretary.*—R. B. Winder, Baltimore.

*Rec. Secretary.*—H. C. Thompson, Washington.

*Rep. Secretary.*—F. F. Drew, Baltimore.

*Treasurer.*—J. Curtiss Smithe, Washington.

*Executive Committee.*—T. S. Waters, Baltimore; M. W. Foster, Baltimore; H. B. Noble, Washington.

This united meeting of the three Associations was arranged with the view of bringing together in social and professional intercourse, to as great an extent as possible, members of the profession from all parts of the country, believing that the best interests of all, both individually and collectively would be thereby promoted. Every effort will be made to have this occasion a pleasant one.

We will have the valuable voluntary co-operation of able and learned members of the Medical Profession, papers will be read from eminent members of the three Associations; our clinical operations will be among the best in the profession—a wider range than usual will be given to the mechanical processes, the battery and the spectroscope will be fully and ably treated in their relations to Dentistry, and if possible the effect of anæsthetics on living organisms will be exhibited.

Deer Park and Oakland, six miles apart, are situated on the backbone of the Alleghany Mountains, immediately on the Baltimore and Ohio Rail Road. By an arrangement with that Company members of the Associations and those desiring to become members will purchase at regular rates, tickets to Deer Park or Oakland from the following or intermediate points, viz: Washington, Baltimore, Pittsburg, Wheeling, Sandusky, Monroeville, Auburn, Chicago, Cincinnati, Columbus, Portsmouth, Parkersburg, Staunton, Lynchburg and Danville. Returning they will receive certificates at Deer Park or Oakland which will pass them over the route they came to the points at which they purchased their tickets.

The Ohio and Mississippi Rail Road will sell to duly accredited members round trip tickets as follows: Louisville to Cincinnati, $5.00, St. Louis to Cincinnati, $12.00. It is believed that other lines connecting with the Baltimore and Ohio will make for this occasion large reduction in the rates of travel.

The Hotels at Deer Park and Oakland can accommodate all who may attend, and the rates will be reduced from $3.50 to $2.50 per day.

The scenery on this Road is grand beyond description, and the mountain air is cool, pure and bracing.

It is earnestly hoped that every member of the Associations will attend, and a cordial invitation to be present is extended to all other Dentists, to members of the Medical Profession and all others interested in Dental affairs.

